# Genomics against gonorrhoea

**DOI:** 10.7554/eLife.59379

**Published:** 2020-06-30

**Authors:** Nicholas Medland

**Affiliations:** Kirby Institute, University of New South WalesSydneyAustralia

**Keywords:** antibiotic resistance, diagnostic, surveillance, *Neisseria gonorrhoeae*, Other

## Abstract

Surveillance strategies based on whole genome sequencing could help with the early identification and detection of new forms of drug-resistant gonorrhoea.

**Related research article** Hicks AL, Kissler SM, Mortimer TD, Ma KC, Taiaroa G, Ashcroft M, Williamson DA, Lipsitch M, Grad YH. 2020. Targeted surveillance strategies for efficient detection of novel antibiotic resistance variants. *eLife* **9**:e56367. doi: 10.7554/eLife.56367

You meet a new sexual partner and a few days later notice genital discharge and discomfort. Distressing. After examination and testing at an STI clinic you receive an antibiotic injection and tablets to treat gonorrhoea. The symptoms disappear. You are reassured that, within a few days, it will no longer be possible for the infection to be transmitted to another person. But how sure can you really be that you are cured? The answer is very sure. But will this always be the case, or might we run out of antibiotics that can treat gonorrhoea?

Today, a patient treated for gonorrhoea can be almost 100% sure of receiving antibiotics with an almost 100% cure rate. This is despite the fact that since the dawn of the antibiotic era, the organism which causes gonorrhoea – a species of bacteria called *Neisseria gonorrhoeae* – has developed resistance to every antibiotic used to treat it. *N. gonorrhoeae* has in turn become resistant to penicillins, tetracyclines, spectinomycin, fluroquinolones and a number of 'third-generation' cephalosporins (Lewis, 2014).

Our ability to keep treating gonorrhoea is due largely to programs in different countries which systematically collect data on antibiotic resistance in *N gonorrhoeae*: when these data show that resistance to the current first-line antibiotic has become widespread, it is replaced with a new antibiotic. However, this situation might not last. First, there is no next-in-line antibiotic ready to take the place of the current first-line drug, a third-generation cephalosporin called ceftriaxone (Lahra et al., 2018). Second, global programs to detect highly drug-resistant strains before they disseminate more widely are inadequate (WHO, 2012).

The global importance of this issue cannot be overstated: gonorrhoea causes an estimated 87 million infections per year (Newman et al., 2015), and the burden of disease is greatest in lower- and middle-income countries and in disadvantaged populations in high-income countries, like Indigenous Australians. Complications include infertility, poor birth outcomes, pelvic inflammatory disease or increased risk of HIV transmission (Mullick et al., 2005). Effective antibiotic treatment is the basis of measures to control gonorrhoea. The global nature of the risk has been highlighted by the detection of highly-resistant infections in travellers returning from countries which do not have strong systems for detecting resistance to countries which do (Lahra et al., 2018).

Resistance testing using bacterial culture is the gold standard. However, despite being low-cost and low-tech, it has been challenging to scale-up culture-based systems in regions with limited laboratory infrastructure (Wi et al., 2017). One reason is that the organism dies rapidly outside the body, so it must be cultured at the bedside or rapidly transported to a specialised laboratory.

Can new technologies overcome some of these obstacles? In 2012, the World Health Organisation called for more research into molecular technologies for antimicrobial resistance surveillance (WHO, 2012). Molecular techniques have some advantages which make them ideal to complement culture-based systems. For example, specimens do not degrade, so they can be transported over long distances by post or stored for later testing. However, there are also disadvantages. One is cost. Culture consumables and equipment are inexpensive and readily available, whereas molecular machines and reagents are expensive and are often patented.

Another disadvantage is that molecular techniques measure the resistance indirectly, whereas culture measures it directly (Low and Unemo, 2016). Culture systems examine the ability of bacteria to grow in the presence of each antibiotic, at escalating concentrations. Molecular techniques, on the other hand, look for genetic sequences known to occur in antibiotic resistant bacteria. Most often these sequences are the genetic mutations which bacteria have developed or acquired to counteract the effects of the particular antibiotic. Testing for such a mutation might involve a genetic probe looking for a highly specific piece of genetic code in a patient sample or, increasingly, sequencing whole genomes and then searching for particular sequences. However, this approach relies on having an up-to-date library of the mutations that confer resistance. It is also necessary to be aware of strains of *N. gonorrhoeae* which might not have been detected by existing molecular diagnostic techniques (a phenomenon known as diagnostic escape).

What is the best way to keep such a molecular library up to date, and to ensure that antibiotic resistance is detected as soon as possible? Analysing every sample every time is not feasible as it would be too expensive and time-consuming. However, antibiotic resistance in gonorrhoea does not occur randomly: could targeted sampling improve discovery of new resistance, and what would be the most efficient way of selecting samples for testing? Now, in eLife, Yonatan Grad and colleagues at Harvard and the University of Melbourne – including Allison Hicks as first author – begin to answer some of these questions (Hicks et al., 2020).

The new study uses five historical datasets of genetic sequences for *N. gonorrhoea*, accompanied by varying amounts of patient, clinical and laboratory information, and compares the performance of different strategies for selecting samples to be tested. The comparisons involved measuring how well the different strategies performed when presented with data that contained known patterns of resistance.

Some of these strategies seem quite intuitive and were based on epidemiological patient characteristics. For example, the researchers found that some targeted sampling, in particular of returned travellers, helps to identify resistant mutants: on the whole, however, this strategy was not that reliable. Other intuitive strategies were based on resistance to other antibiotics. A different type of strategy used the recorded genetic sequence of the bacteria, in particular measures of diversity and relatedness of difference strains. Overall strategies guided by genomics were significantly more efficient in identifying genetic variants associated with resistance and diagnostic escape. The challenge is to efficiently harness the power of these new methods so that they can complement global public health programs fighting antibiotic resistance in gonorrhoea.
